# Corticotropin‐releasing factor type 1 receptors in the rat nodose ganglion are involved in the transduction of stress‐induced visceral sensory signals to the brain

**DOI:** 10.1111/jne.70082

**Published:** 2025-08-21

**Authors:** Asuka Mano‐Otagiri, Tamotsu Shibasaki, Atsushi Sakai, Yoshihiko Kakinuma

**Affiliations:** ^1^ Department of Bioregulatory Science Nippon Medical School Tokyo Japan; ^2^ Nippon Medical School Tokyo Japan; ^3^ Department of Pharmacology Nippon Medical School Tokyo Japan

**Keywords:** corticotropin‐releasing factor type 1 receptor, immunoreactivity, nodose ganglion, stresscolon

## Abstract

Corticotropin‐releasing factor (CRF) plays roles in stress‐related responses through its type 1 (CRF_1_) and type 2 receptors. Both CRF and CRF_1_ are expressed in the rat colon. Peripheral CRF administration and various stressors increase colonic motility and defecation. Stress induces CRF release in the colon, suggesting CRF may mediate stress‐related responses of the colon. The vagal nodose ganglion (NG) transduces visceral information, including colonic sensation, to the brain. However, it remains unclear whether the CRF/CRF_1_ system is involved in vagal afferent functions. This study, therefore, aimed to clarify the involvement of the CRF/CRF_1_ system in relaying visceral sensory information to the brain and the effect of stress exposure on vagal nerve function. The experiments were conducted in male rats. First, CRF_1_‐like immunoreactivity (CRF_1_‐LI) was characterized in the NG. Second, the effects of vagotomy on CRF_1_‐LI in the NG, intraperitoneally administered CRF‐induced fecal output, and c‐Fos expression in the nucleus tractus solitarius (NTS) were evaluated. Subsequently, a fast blue retrograde tracer was microinjected into the proximal colon. Finally, we analyzed CRF‐ or stress‐induced phosphorylation of cyclic AMP‐response element‐binding protein (pCREB) in the NG. CRF_1_ mRNA and CRF_1_‐LI were detected, and CRF_1_‐LI accumulated on the proximal side of the ligated region of the nerve trunk, and CRF_1_‐LI was detected in most cholinergic neurons. CRF_1_ siRNA suppressed the expression of CRF_1_‐LI in the NG. Subdiaphragmatic vagotomy decreased the number of CRF_1_‐positive cells in the NG while it did not affect CRF‐induced fecal output. CRF‐induced c‐Fos expression in the NTS was suppressed by vagotomy. A neuronal tracing study showed that approximately half of CRF_1_‐positive cells expressed fast blue in the NG. Intraperitoneal CRF, a selective CRF_1_ agonist, or immobilization stress induced pCREB expression and increases in CRF_1_‐positive cells in the NG. In contrast, a CRF_1_ antagonist reduced the immobilization‐induced increase in the expression of pCREB in the NG. These results suggest that the CRF/CRF_1_ system is involved in the signal transduction of colonic sensory information to the central nervous system via the NG.

## Graphical abstract

Corticotropin‐releasing factor (CRF) and its type 1 receptor (CRF_1_) are expressed in the rat colon. The vagal nodose ganglion transmits visceral information to the brain; but whether the CRF/CRF_1_ system is involved in vagal afferent function remains unknown. This study confirmed the role of CRF/CRF_1_ in mediating colonic sensory information to the central nervous system via the nodose ganglion in Wistar rats.

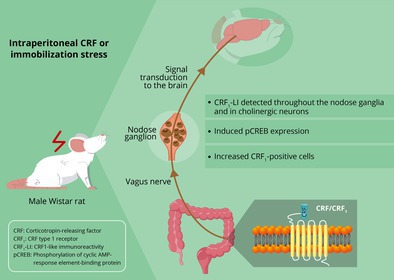

## 1 INTRODUCTION

Corticotropin‐releasing factor (CRF) is a 41‐amino‐acid peptide that was initially identified as a hypothalamic factor that stimulates the secretion of pituitary adrenocorticotropic hormone and mediates endocrine, behavioral, and autonomic responses to stress.^1^ CRF actions are mediated by two distinct receptor subtypes, CRF type 1 (CRF_1_) and CRF type 2 (CRF_2_), which belong to the class B subfamily of seven transmembrane G‐protein‐coupled receptors.^2^

Recent studies have reported that CRF and its receptors are expressed in the rat colon.^3^, ^4^ In the proximal and distal colon, CRF is largely detected in enterochromaffin cells and enteric neurons; it is also observed that stress increases CRF expression in these cells.^3^ CRF_1_ and CRF_2_ are detected in goblet cells in the colonic mucosa, scattered cells of the absorptive surface epithelium, isolated cells in the lamina propria, and neurons in the submucosa and muscularis.^4^

Peripheral CRF administration is known to increase colonic motility and fecal output; thus, CRF may play an important role in promoting local colonic functions.^5^, ^6^ Various stressors increase colonic motility, permeability, and fecal output.^7^, ^8^, ^9^ Peripherally injected selective CRF_1_ antagonists suppress the increase in colonic motility induced by acute stress or peripheral injection of CRF.^7^, ^10^ Intraperitoneal injection of urocortin 2 (Ucn 2) activates peripheral CRF_2_ and reduces defecation and colonic motility induced by acute stress or CRF in mice; whereas intraperitoneal injection of astressin2‐B, a peptide CRF_2_ antagonist, increases stress‐induced colonic motility.^11^ These investigations suggest that the colonic motor responses are directly initiated in the colon through CRF_1_ activation by its ligands. In contrast, Ucn 2 inhibits the colonic motor responses via CRF_2_.

In the rat colon, stress increases the expression of CRF mRNA and protein in the myenteric and submucosal plexuses, and partial restrained stress increases CRF release.^9^ Peripheral CRF administration increases the expression of the c‐Fos protein, a marker of neuronal activity, in intrinsic neurons of the myenteric ganglia of the proximal colon of rats, and CP‐154, 526, a selective CRF_1_ antagonist, blocks CRF‐induced Fos expression.^12^ These investigations suggest that CRF possibly binds to CRF_1_ located in neighboring neurons and activates neurons in the myenteric ganglia, which are involved in colonic motility.^12^

Signal transduction to the central nervous system from the gastrointestinal tract involves two main pathways: vagal afferent neurons and spinal afferent neurons.^13^ Sensory information is transduced by nerve endings that inform the brain. The nerve endings of vagal afferent neurons located in the visceral organs transmit visceral sensory information to the brainstem via the nodose ganglion (NG).^13^ Vagal afferent neurons respond directly to mechanical, chemical, or inflammatory stimuli, whereas spinal afferent neurons serving the gastrointestinal tract are primarily involved in the transmission of noxious stimuli.^13^, ^14^ Recently, an RNA sequencing study revealed that CRF_2_ mRNA but not CRF_1_ mRNA is detected in the rat NG.^15^ The function of CRF_2_ in the NG is not clear; however, the anorexigenic effect of urocortin 1 was blocked by the central CRF_2_ antagonist^16^, ^17^ and the CRF_2_ antagonist K41498 binds to the vagal afferent terminal in the nucleus tractus solitarius (NTS).^18^ Therefore, CRF_2_ may be transported to the presynaptic terminal in the brain from the cell body in the NG. Although described above, the expression of CRF_1_ mRNA is not detected using RNA sequencing study, it is reported that ovine CRF, which has an affinity higher for CRF_1_ rather than CRF_2_, binds to the sites controlling axonal transport in the rat vagus nerve.^19^ It is possible that the CRF_1_ are involved in the transduction of visceral information to the brain.

Previously, we generated an anti‐CRF_1_ antiserum against a synthetic peptide (SPEVHQSNVAWC) corresponding to amino acid residues 177–188 of the first extracellular loop of rat CRF_1_, which shares 42% homology with CRF_2α_, as described previously.^20^ We investigated the distribution of CRF_1_‐like immunoreactivity (LI) and the characteristics of CRF_1_‐LI‐expressing cells, and evaluated the effect of glucocorticoids on CRF_1_‐LI in the rat pituitary. Western blot analysis revealed that the anti‐CRF_1_ antiserum recognized CRF_1_ but not CRF_2_ in experiments using CRF_1_‐ or CRF_2_‐overexpressing HEK‐293 cells and the suppressive effect on CRF_1_‐LI of siRNA against CRF_1_ but not against CRF_2_ in the primary cultured anterior pituitary cells.^20^

In the present study, we therefore conducted several experiments to clarify the function of the CRF/CRF_1_ system in the NG in signal transduction of visceral sensory information to the central nervous system in the rat. First, CRF_1_‐LI was characterized in the rat NG. Second, the effects of subdiaphragmatic vagotomy on CRF_1_‐LI in the NG, intraperitoneally administered CRF‐induced fecal output, and c‐Fos expression in the NTS were evaluated. Subsequently, a fast blue retrograde tracer was microinjected into the proximal colon to determine the colonic innervation patterns involving vagal afferents. Finally, we analyzed CRF‐ or stress‐induced phosphorylation of cyclic AMP‐response element‐binding protein (pCREB) in the NG.

## 2 MATERIALS AND METHODS

### 2.1 Animals

Male Wistar rats (Tokyo Laboratory Animals, Science Co., Ltd., Tokyo, Japan) weighing 200–230 g were housed under controlled illumination (12‐h light–dark cycle starting at 08:00), humidity, and temperature (24°C), with free access to laboratory chow and water. The number of rats used in the experiment was 290 in total. Bilateral NG were used in all the experiments. In the female rats, the estrus cycle influences hypothalamic‐pituitary‐adrenal axis responsiveness to stress, as we have previously reported,^21^ we conducted the experiment using only male rats in the present study. To circumvent the circadian influence, all functional studies were started at 9 in the morning, and rats were fed status to exclude the fasting effects.

CRF_1_‐immunoreactivity was expressed in both sides of NG, and both the left and right vagus nerves project to the colon; then, bilateral NGs were used in the experiment and no distinction was made between the left and right.

All experimental procedures were approved by the Laboratory Animal Ethics Committee of Nippon Medical School.

### 2.2 Real‐time quantitative polymerase chain reaction (qPCR) analysis

Total RNA from the four NGs was extracted using ISOGEN II (Nippon gene Co., Ltd., Tokyo, Japan) following the protocol provided by the manufacturer. cDNA was reverse‐transcribed with ReverTra Ace qPCR RT Master Mix (Toyobo Life Science, Osaka, Japan), followed by qPCR with GoTaq qPCR Master Mix (Promega KK, Tokyo, Japan) using Thermal Cycler Dice Real Time System TP800 (Takara Bio Inc., Shiga, Japan). The primers of genes (Takara Bio) were rat CRF_1_ transcript variant 1 (NM_030999.5, forward: 5′‐CCCATGATCCTGGTCCTGCT‐3′, reverse: 5′‐GATGTAGTGGATGCCCGGAGTT‐3′) and rat CRF_2_ (NM_022714.2, forward: 5′‐AATGGGACCTGGGCCTCAA‐3′, reverse: 5′‐GGAAACACAGTGGCCCAGGTA‐3′). GAPDH (NM_017008.4, forward: 5′‐GGCACAGTCAAGGCTGAGAATG‐3′, reverse: 5′‐ATGGTGGTGAAGACGCCAGTA‐3′) was used as a housekeeping gene to normalize target gene transcript levels.

### 2.3 Western blot analysis

According to previous studies,^20^, ^22^ 10 μg of protein in each lane, which was isolated from six NG or whole pituitaries of rats, was mixed with a sampling buffer (0.05 M Tris–HCl pH 6.8, 0.1 M DTT, 0.07 M SDS, 0.1% [W/V], bromophenol blue, 6% (v/v) glycerol, and 2.5% (v/v) 2‐mercaptoethanol), and comparably electrophoresed in a 12.5% sodium dodecyl‐sulfate polyacrylamide electrophoresis gel and blotted onto a polyvinylidene difluoride membrane. It was reacted with a primary antibody, anti‐CRF_1_ antiserum, followed by a horse radish peroxidase‐conjugated secondary antibody, and signals were detected with a C‐DiGit Blot Scanner (LI‐COR Corp., Lincoln, NE) using an ImmunoStar LD (Wako Pure Chemical Industries, Ltd., Tokyo, Japan).

### 2.4 Immunohistochemistry

Rats were sacrificed by intraperitoneal injection of an overdose of sodium pentobarbital (100 mg/kg body weight) and xylazine (10 mg/kg body weight) and transcardially perfused with 4% paraformaldehyde solution in 0.1 M phosphate‐buffered solution (PBS, pH 7.4). NG was removed and processed for immunohistochemistry as described previously.^20^ After being post‐fixed in paraformaldehyde overnight, the NG were placed in 20% sucrose in PBS and then cut into 14‐μm thick longitudinal sections using a cryostat. The sections were mounted on glass slides and stored at −80°C until immunohistochemical processing.

To neutralize endogenous peroxidase, the sections were incubated with 0.3% H_2_O_2_ in methanol for 30 min. Subsequently, non‐specific sites were blocked with a blocking solution (3% normal donkey serum in PBS; Jakson ImmunoResearch Inc., PA) for 20 min. The anti‐CRF_1_ antiserum, with or without pretreatment, and the antigenic peptide (10 μg/mL) 177–188 amino acid fragment of the first extracellular loop of the CRF_1_ were applied (1:500) in PBS and then incubated overnight at 4°C. To further evaluate the specificity of anti‐CRF_1_ antiserum, the antiserum that had been pretreated with the excess amount of the 173–184 amino acid fragment of the first extracellular loop of the CRF_2_α (100 μg/mL), which corresponds to the same portion of the CRF_1_ antigenic peptides, was also used (1:500) as described above. Sections were incubated for 30 min in a blocking solution containing biotinylated donkey anti‐rabbit IgG (1:200; Jakson ImmunoResearch Inc.) and processed in an avidin–biotin–peroxidase complex solution (ABC Vectastain Elite Kit, Vector Laboratories, Inc., Burlingame, CA) for 30 min. The antibody–peroxidase complex was visualized using 3,3′‐diaminobenzidine hydrochloride (Vector DAB Kit; Vector Laboratories, Inc.). When staining reached the appropriate intensity, the tissue was rinsed in PBS, dehydrated through graded alcohol series, cleared in xylene, and coverslipped with VectaMount (Vector Laboratories, Inc.). Images were captured using a microscope (BX‐51; Olympus, Tokyo, Japan) equipped with a digital image analysis system (CellSens Standard; Olympus).

### 2.5 Calculation of the cell number in the NG

Since the size of NG neurons is less than 80 μm, the whole NG was cut into 6–10 sagittal cryosections with 14‐μm thickness at 84‐μm intervals to minimize the double counting of cells, and the labeled cells were counted.

Other sets of every six sections were counterstained with 0.1% cresyl violet, and the other sets of every six sections were examined using the anti‐CRF_1_ antiserum and dehydrated with graded ethanol and cover‐slipped. The total number of NG neurons was calculated, and positive cells were counted by an observer blinded to the treatment conditions of each section.

### 2.6 Vagal transport of CRF_1_ using a nerve trunk ligation study

The vagal nerve was ligated to evaluate vagal transport of the receptors. The rats were anesthetized intraperitoneally with sodium pentobarbital (50 mg/kg body weight) and xylazine (10 mg/kg body weight). A sagittal cervical incision was made in the left anterior neck, and a ligature was placed on the cervical vagal nerve fibers 2 mm distal to the NG for 4 h; as reported previously.^23^ After 4 h, the animals were sacrificed using an overdose of sodium pentobarbital and xylazine and processed using paraformaldehyde as described above. Next, the NG was sectioned for immunohistochemistry using anti‐CRF_1_ antiserum.

### 2.7 Validation of the anti‐CRF_1_ antiserum through immunohistochemistry

To evaluate the specificity of the anti‐CRF_1_ antiserum, dual‐immunofluorescence labeling was performed using an anti‐CRF receptor goat polyclonal antiserum, which was prepared by immunization with an antigen, the amino acid fragment of the C‐terminus of human CRF_1_ (C‐20; Santa Cruz Biotechnology Inc., West Grove, CA). NG sections were incubated with the anti‐CRF_1_ serum (1:500) overnight at 4°C, rinsed in PBS, and incubated in Alexa Fluor 488‐labeled donkey anti‐rabbit IgG (1:200; Jakson ImmunoResearch Laboratories Inc.) for 3 h at room temperature. Then, the sections were washed in PBS, subsequently incubated with the anti‐CRF receptor antiserum (C‐20; 1:500) overnight at 4°C, rinsed in PBS, and incubated in Alexa Fluor 647‐labeled donkey anti‐goat IgG (1:200; Jakson ImmunoResearch Laboratories Inc.) for 3 h at room temperature. The slides were cover‐slipped with VECTASHIELD mounting medium (Vector Laboratories Inc.).

In situ hybridization was conducted to confirm the expression of CRF_1_ mRNA in rat NG. Digoxygenin‐labeled antisense and sense (control) cRNA copies were synthesized from the full‐length (1.3 kb, a gift from Dr. W Vale) rat CRF_1_ cRNA^24^, ^25^ and subcloned into a pBluescript SK‐I transcription vector. Samples were digested with digoxygenin‐UTP and T7 polymerase for 120 min at 37°C (Roche Diagnostics GmbH, Basel, Switzerland). Tissues were sectioned on a cryostat maintained at −20°C, mounted onto poly‐l‐lysine‐coated slides, and then digested with 1 μg/mL of proteinase K for 30 min at 37°C. For fixation, sections were placed in 4% PFA at room temperature. After 10 min, the sections were subjected to successive washes in 0.1 M triethanolamine, pH 8.0/0.25% acetic anhydride for 10 min and PBS for 5 min twice. The sections were dehydrated using a graded alcohol series and air dried. RNA probes were used at a concentration of approximately 1 ng/mL and applied to sections in a hybridization buffer containing 50% formamide, 5× saline sodium citrate (SSC) buffer, 0.025% tRNA, 5× Denhardt's solution, 10% dextran sulfate, and salmon sperm and hybridized overnight at 72°C. After hybridization, the sections were washed twice in 50% formamide, 2× SSC for 15 min, treated with 20 μg/mL of ribonuclease A for 60 min at 37°C, and washed twice in 2× SSC at 72°C and 0.5× SSC for 15 min at 72°C. After rinsing, the sections were treated with an anti‐digoxygenin fluorescein‐conjugated antibody (Roche Diagnostics GmbH) diluted 1:2000 for 3 h at room temperature, and the slides were air‐dried and cover‐slipped with VECTASHIELD‐mounting medium. Immunofluorescence in tissue sections was visualized using a Leica TCS SP5 confocal microscope (Leica Microsystems, Wetzlar, Germany) with a multiband filter set for the independent or simultaneous visualization of fluorescein (absorption maximum, 494 nm; emission maximum, 519 nm). The specificity of the probe was confirmed by the absence of signals in sections labeled with the sense probe.

### 2.8 Quantification of both CRF_1_ ‐ and choline acetyltransferase (ChAT)‐positive cells in the NG

To characterize the CRF_1_‐expressing cells, dual‐immunofluorescence labeling was performed using anti‐CRF_1_ antiserum and anti‐ChAT. Polyclonal goat anti‐ChAT antiserum (Millipore Cat no. AB144P, RRID: AB 2079751, San Diego, CA) was used as a marker for cholinergic neurons at a concentration of 1:200. Then, sections were incubated with anti‐CRF_1_ antiserum overnight at 4°C. After being washed with PBS, the tissues were incubated in Alexa Fluor 488‐labeled donkey anti‐rabbit IgG (Jakson ImmunoResearch, Inc.) for 3 h at room temperature, respectively. The sections were then washed in PBS, incubated with the anti‐ChAT antiserum overnight at 4°C, rinsed in PBS, and incubated in Alexa Fluor 647‐labeled donkey anti‐goat IgG (Jakson ImmunoResearch, Inc.) for 3 h at room temperature. The slides were cover‐slipped with VECTASHIELD mounting medium (Vector Laboratories Inc.).

Immunofluorescence in tissue sections was visualized using a Leica TCS SP5 confocal microscope with a multiband filter set for independent or simultaneous visualization of Alexa Fluor® 488 (absorption maximum, 496 nm; emission maximum, 519 nm) and Alexa Fluor 647 (absorption maximum, 651 nm; emission maximum, 667 nm) fluorophores. Images were captured at 512 × 512 pixels using a ×40 objective using immersion oil. The percentage of dual‐labeled cells for cholinergic neurons was based on the average number of four rats.

### 2.9 Small interference RNA

CRF_1_ (MISSION siRNA SASI_Rn01_00083772) and CRF_2_ (MISSION siRNA SASI_Hs01_00014313) siRNA and control siRNA (MISSION siRNA Universal Negative Control no. 1) were purchased from Sigma‐Aldrich‐Japan (Tokyo, Japan). Rats were anesthetized with a mixture of midazolam (0.075 mg/kg), medetomidine (0.4 mg/kg), and butorphanol (0.5 mg/kg), administered intraperitoneally and placed in a supine position on a custom‐made surgical plate. The left NG was exposed by way of a ventral approach. The incision was made from the middle of the neck. With the surgical operating microscope, the caudal end of the ganglion attached by the vagal nerve was separated from the carotid artery. A 10 μL glass syringe affixed with a custom‐made 33‐gauge needle was filled with siRNA (2 μg/2 μL) dissolved in distilled water. When siRNAs were injected into the left NG, the needle was left in the ganglion for 10 min. The incision was closed and, 3 days later, rats were perfused, and siRNA‐injected left NGs were processed for immunohistochemistry described above. Since the injection of siRNA to the right side of the NG increased the mortality rate, we administered it to the left side of the NG alone. The number of CRF_1_ positive cells was measured using confocal microscopy (SP5, Leica) with excitation at 647 nm. The number of CRF_1_‐positive cells was counted and subjected to statistical analysis.

### 2.10 Subdiaphragmatic vagotomy

To determine the effects of vagotomy on: (i) the number of CRF_1_‐positive cells in the rat NG, (ii) CRF‐induced fecal output; and (iii) c‐Fos expression (a marker of neuronal activity), in the NTS of the brainstem, a subdiaphragmatic truncal vagotomy 1 cm proximal to the esophagogastric junction (or a sham operation as a control)^26^, ^27^ was performed 7 days before intraperitoneal CRF injection. After a midline incision in the abdominal wall, the lower part of the esophagus was exposed, and the anterior and posterior branches of the vagal nerve were incised under anesthesia with intraperitoneal injection of sodium pentobarbital (50 mg/kg body weight) and xylazine (10 mg/kg body weight), as described previously.^26,^, ^27^ During sham operations, the vagal trunks were similarly exposed without cutting the vagal nerve.

Seven days after the operation, sham‐operated (*n* = 6) and vagotomized (*n* = 6) rats were sacrificed with an overdose of sodium pentobarbital and xylazine and processed using paraformaldehyde as described above. As described above, the NG was sectioned for immunohistochemistry using an anti‐CRF_1_ antiserum, and then the number of CRF_1_‐positive cells was counted.

On the day of the experiment, rats were divided into four groups: sham‐operated rats injected with saline (*n* = 8), sham‐operated rats that received an intraperitoneal CRF injection (10 μg/kg body weight) (*n* = 7), vagotomized rats injected with saline (*n* = 8), and vagotomized rats that received an intraperitoneal CRF injection (10 μg/kg body weight) (*n* = 8). CRF was purchased from Peptide Institute (Osaka, Japan) and the dose enhancing the colonic motor function was determined as previously reported.^7^ After 1 h, the number of fecal samples was counted, and after 3 h, the animals were sacrificed with an overdose of sodium pentobarbital and xylazine. The brains were processed using paraformaldehyde, as described above. Twenty serial coronal sections were cut into 40 μm sections through the NTS. Immunohistochemical analysis was performed using the free‐floating ABC method with a c‐Fos antibody (1:10,000; rabbit polyclonal, Ab5; Oncogene, San Diego, CA). The antibody–peroxidase complex was visualized using diaminobenzidine (Vector DAB Kit, Vector Laboratories). The levels of Fos expression in the NTS and the number of CRF_1_‐expressing cells in the NG were quantified. We have performed the quantification in the NTS bilaterally, referring to the atlas of Paxinos and Watson.^28^

### 2.11 Retrograde tracing and axonal transport

Four rats were anesthetized using intraperitoneal sodium pentobarbital (50 mg/kg body weight) and xylazine (10 mg/kg body weight). Using aseptic techniques, after a middle incision in the abdominal wall, a suspension of 2.5% fast blue (FB; Polysciences, Inc., Warrington, PA) in distilled water was injected into the proximal colon from a site 1 cm distal to the cecocolic junction within 5 cm at 10 sites (1 μL each), using a 10‐μL 30 G Hamilton microsyringe. The FB concentration was determined based on the manufacturer's instructions. After each injection, the needle was left in place for up to 1 min to reduce dye leakage. The microinjection procedure has been previously described.^29^ The viscera were placed in the abdominal cavity, and the incision in the abdominal muscle and skin was sutured. The animals were administered antibiotics, Procaine Penicillin G (10,000 unit/kg, intraperitoneally; Nippon Zenyaku Kogyo Co., Ltd., Fukushima, Japan).

Nine days after the injection, the rats were anesthetized with an overdose of sodium pentobarbital (100 mg/kg body weight) and xylazine (10 mg/kg body weight); and transcardially perfused with PBS followed by 4% paraformaldehyde.

NG was removed, incubated overnight in 4% paraformaldehyde, and transferred to 20% sucrose/PBS. Sections (14 μm) were cut along the long axis and processed for dual immunofluorescence of FB on CRF_1_‐positive cells. Sections were immunostained with anti‐CRF_1_‐antiserum and Alexa Fluor 647‐labeled goat anti‐rabbit IgG, and labeled cells were observed using a Leica TCS SP5 confocal microscope with a filter set for FB (excitation wavelength, 365 nm; emission wavelength, 420 nm) and Alexa Fluor 647. Every six sections of the NG, fluorosphere‐positive neurons were counted.

### 2.12 Quantitating intraperitoneal CRF administration or stress‐induced changes in pCREB expression in the NG

In this study, we examined the expression of pCREB in the NG to reveal whether vagal afferent neurons respond to immobilization stress and intraperitoneal CRF injection. Immobilization stress was performed by wrapping rats with a wire‐mesh sheet to prevent them from moving. Rats received immobilization stress for 60 min or intraperitoneal CRF injection (10 μg/kg body weight) without anesthesia; they were then perfused with 4% paraformaldehyde 3 h after treatment under anesthesia described previously. The NG was removed and immunohistochemically processed. pCREB was detected by affinity‐purified rabbit monoclonal phospho‐CREB (Ser133) (87G3) antibody (1:500, Cell Signaling, Cat no. 9198, RRID: AB_2561044, Danvers, MA) with secondary antibody and donkey anti‐rabbit IgG conjugated to Alexa Fluor 488 (Jackson ImmunoResearch, Inc.). Because the primary antibody often cross‐reacts with IgGs raised in the same host, we used CRF_1_ antibody raised in goat (C‐20, Santa Cruz Biotechnology Inc.) described above to avoid detection of false colocalization signals. The other set of slides in the same NG for dual immunofluorescence was incubated with the anti‐CRF_1_ antibody (C‐20) with secondary antibody, donkey anti‐goat IgG conjugated to Alexa Fluor 647 (Jackson ImmunoResearch, Inc.), phospho‐CREB (Ser133) (87G3) antibody (1:500, Cell Signaling) with secondary antibody, and donkey anti‐rabbit IgG conjugated to Alexa Fluor 488 (Jackson ImmunoResearch, Inc.). Slides were examined under a Leica TCS SP5 confocal microscope, and labeled cells were observed with a filter set for Alexa Fluor 488 and Alexa Fluor 647. The pCREB‐positive neurons were counted in the NG.

### 2.13 The effect of CRF_1_ agonist, Ucn 2, CRF_1_ antagonist, and CRF_2_ antagonist on the stress‐induced pCREB expression in the NG

Cortagine (10 μg/kg, Med Chem Express, NJ), CRF_1_ agonist, or vehicle (10% dimethyl sulfoxide; DEMSO, FUJIFILM Wako Pure Chemical corporation, Tokyo, Japan) was injected intraperitoneally, and rats were perfused 3 h after injection and processed for immunohistochemistry as described above. Since intraperitoneal injection of 10 μg/kg cortagine is reported to activate colonic motor function,^30^ we used the same dose in the experiment. Ucn 2, an endogenous ligand for CRF_2_ or saline was also injected intraperitoneally, and rats were processed for dual‐immunohistochemistry of pCREB and CRF_1_.

To determine the role of CRF_1_ in the stress‐induced expression of pCREB in the NG, antalarmin (20 mg/kg), a selective antagonist of CRF_1_, or vehicle (10%cremaphor EL, Sigma‐Aldrich; 5% ethanol; 85% H_2_O) was injected intraperitoneally 30 min before immobilization stress. The dose of antalarmin is shown to abolish colonic contraction in response to CRF administration.^31^ In addition, to ascertain the role of CRF_2_ on the stress‐induced expression of pCREB in the NG, antisauvagine‐30 (100 μg/kg), an antagonist of CRF_2_, or saline was injected intraperitoneally 30 min before immobilization stress. Based on previous reports, we selected the dosage of antisauvagine‐30.^10,^, ^11^ Rats were perfused 3 h after the onset of immobilization stress and processed for dual‐immunohistochemistry of pCREB and CRF_1_. The slides were examined under a Leica TCS SP5 confocal microscope, and labeled cells were detected with a filter set for Alexa Fluor 488 and Alexa Fluor 647. In all sections at 84 μm intervals, pCREB‐positive neurons were counted in the NG.

### 2.14 Statistical analysis

All results are expressed as mean ± SEM. Data on the effect of vagotomy on the expression of CRF_1_‐positive cells, the expression of CRF‐ or stress‐induced pCREB, CRF_1_, dual‐positive cells, pCREB or CRF_1_ on dual‐positive cells, and the expression of pCREB, CRF_1_, double‐positive cells pretreated with cortagine, Ucn 2, and vehicle were analyzed using the unpaired *T*‐test. Data obtained from the number of CRF_1_‐positive cells in the rats NG injected with siRNA were subjected to one‐way analysis of variance, with siRNA as factors. Data obtained from fecal output and Fos expression in CRF‐injected vagotomized rats were subjected to two‐way analysis of variance, with CRF injection and surgery as factors. Data obtained from antalarmin‐ or vehicle‐injected stressed rats or naive rats were subjected to two‐way analysis of variance, with antalarmin injection and stress as factors. In the group subjected to one‐ or two‐way analysis of variance, subsequently, the Bonferroni–Dunn test was used for multiple comparisons. All data are expressed as means ± SEM. Statistical analysis was performed using StatView 4.5 (Abacus Concepts, Inc., Berkeley, CA). The significance level was set at *p* < .05.

## 3 RESULTS

### 3.1 RT‐qPCR of CRF_1_ and CRF_2_ in the rat NG

**FIGURE 1:**
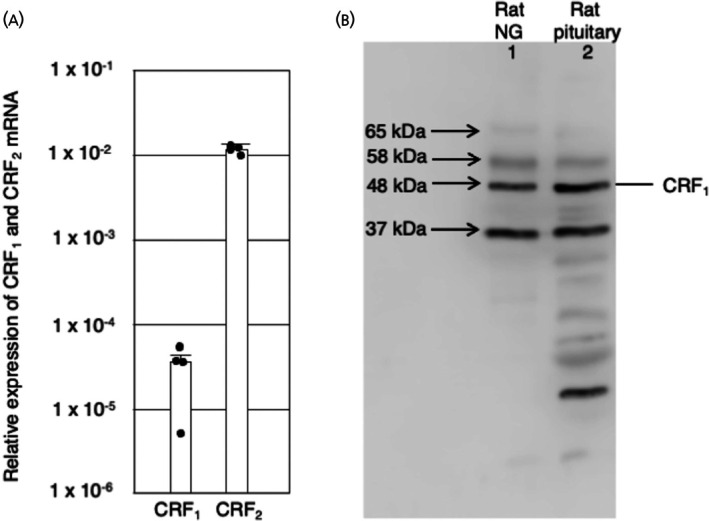
Expression of corticotropin‐releasing factor receptor type 1 (CRF_1_) in the nodose ganglion (NG). (A) Reverse‐transcriptase polymerase chain reaction RT‐PCR analysis for CRF_1_ and corticotropin‐releasing factor receptor type 2 (CRF_2_) in NG. RT‐pPCR Analysis of real time quantitative PCR using comparative Cycle threshold (Ct) method was shown. For each sample, the relative expression level was normalized by the level of glyceraldehyde‐3‐phosphate dehydrogenase. Data are presented as the mean ± SEM. (B) Western blot analysis of the protein extract from the rat NG (lane 1) and pituitary (lane 2) using anti‐corticotropin‐releasing factor receptor type 1 antiserum. The molecular weight of the bands was indicated by an arrow in the left side of the panel. The size of the marker was CRF_1_ in the right side of the panel.

RT‐qPCR from rat NG revealed that the expression of CRF_1_ transcript variant 1 mRNA was much lower than that of CRF_2_ mRNA (Figure 1A).

### 3.2 Western blot analysis of CRF_1_ from rat NG

Western blot analysis of tissue extracts from rat NG using the anti‐CRF_1_ antiserum showed four bands at 65, 58, 48, and 37 kDa, although the expression level of the 58‐ and 65‐kDa bands was weaker than those of the 48‐ and 37‐kDa bands (Figure 1B, lane 1). The pattern of bands obtained from the rat NG was similar to that of the rat pituitary gland (Figure 1B, lane 2).

### 3.3 Immunohistochemical localization and validation of CRF_1_ ‐LI

**FIGURE 2:**
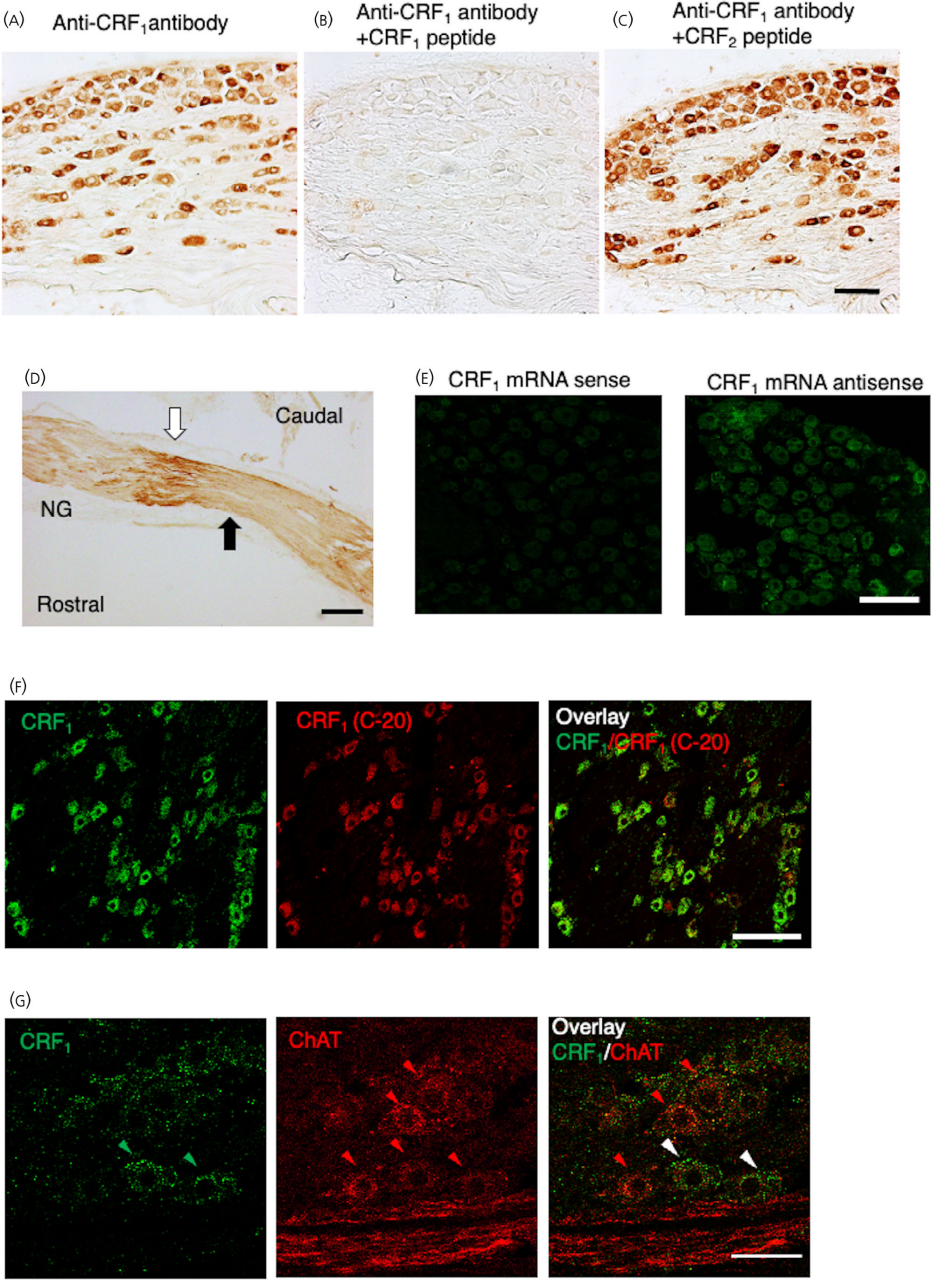
Immunohistochemical analysis of corticotropin‐releasing factor receptor type 1 (CRF_1_)‐like immunoreactivity (CRF_1_‐LI) in the rat nodose ganglion (NG). Using anti‐CRF_1_ antiserum without (A) or with its antigenic peptide, the fragment of 177–188 amino acid residues of the first extracellular loop of CRF_1_ (B) or with the fragment of 173–184 amino acid residues of the first extraloop of the corticotorpin‐releasing factor type 2 receptor (CRF_2_) corresponding to the 177–188 amino acid residues of the first loop of CRF_1_ (C). Scale bar = 100 μm. (D) Nerve trunk ligation of the rat vagal nerve on the peripheral side of the NG. The accumulated CRF_1_‐LI in the rostral side of a ligature is indicated by a white arrow, and the ligation site of the vagal afferent is indicated by a black arrow. Scale bar = 200 μm. (E) Distribution of CRF_1_ mRNA in the rat NG with sense probes (left panel) and antisense probes (right panel). Scale bar = 100 μm. (F) Dual immunofluorescence of CRF_1_‐LI using both anti‐CRF_1_ antiserum (left panel, green) and commonly used antiserum (center panel, C‐20). Overlay image (right panel). Scale bar = 100 μm. (G) Dual‐immunofluorescence staining of CRF_1_‐LI and choline acetyl transferase (ChAT) in the NG. CRF_1_‐LI; left panel, green, ChAT‐positive cells; center panel, red and overlay image; right panel. The white arrowhead indicates the colocalization of CRF_1_‐LI and ChAT. The green arrowhead indicates CRF_1_‐LI and the red arrowhead indicates ChAT‐positive cells. Scale bar = 100 μm.

A positively stained neuronal cell soma was observed throughout the rat NG (Figure 2A). CRF_1_‐LI was not detected when the antiserum was pre‐treated with its antigenic peptide (Figure 2B); whereas CRF_1_‐LI was not affected when the antiserum was pre‐treated with the 173–184 amino acid residue fragment of rat CRF_2_ corresponding to the 177–188 amino acid residue fragment of the first extracellular loop of rat CRF_1_ (Figure 2C). The number of CRF_1_‐positive neurons in the NG was 184 ± 18 (*n* = 20) and the total number of cresyl violet‐positive neurons in the NG was 1276.5 ± 97.6 (*n* = 24), so the rate of CRF_1_‐positive neurons in the NG was 15.5% ± 1.3%.

To determine whether CRF_1_‐LI was transported to the peripheral sensory terminals, NG axons were ligated. CRF_1_‐LI accumulation was found on the proximal side of the 4 h‐nerve trunk ligation site, while CRF_1_‐LI accumulation was unclear on the distal side of the 4 h‐nerve trunk ligation site (Figure 2D).

The distribution of CRF_1_ mRNA in the NG showed a widespread pattern when antisense probes were used; while the labeled sense strand cRNAs failed to show positive localization (Figure 2E).

Dual immunofluorescence analysis using both the anti‐CRF_1_ antiserum and the commonly used antiserum (C‐20) showed 94.5% ± 1.8% similarity (double‐positive cells in anti‐CRF_1_ antiserum‐labeled cells). The results are almost the same (Figure 2F).

The dual‐immunofluorescence studies also showed that ChAT was expressed in 92.0% ± 3.6% of CRF_1_‐positive cells in the NG, whereas 44.9% ± 3.8% (*n* = 4) of ChAT‐positive cells expressed CRF_1_ (Figure 2J).

### 3.4 Suppressive effect of CRF_1_ ‐specific siRNA on the expression of CRF_1_ ‐positive cells

**FIGURE 3:**
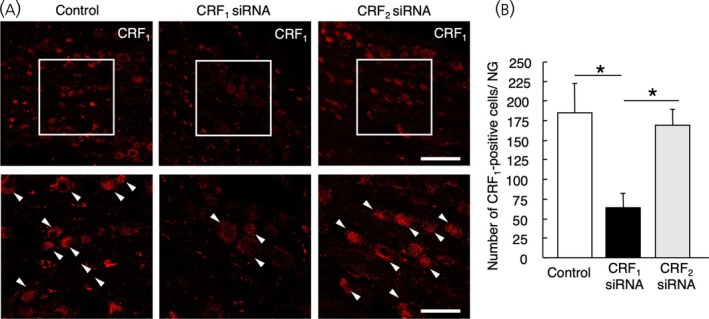
Effects of small interference RNA on the corticotropin‐releasing factor type 1 receptor‐like immunoreactivity (CRF_1_‐LI) in the nodose ganglion (NG). (A) Representative images of the immunohistochemical analysis of CRF_1_‐LI in the NG treated with microinjected universal control siRNA (left panel), CRF_1_ siRNA (center panel), CRF_2_ siRNA (right panel). White arrowheads indicate CRF_1_‐positive cells. Scale bar (upper panel) = 100 μm. The lower panels indicate high magnification of the boxed area in the upper panels, respectively. Scale bar = 50 μm. Data in (B) are presented as means ± SEM. *p < .01 when compared with CRF_1_ siRNA injected NG.

Microinjection of CRF_1_ siRNA significantly suppressed the expression of CRF_1_‐positive cells; however, CRF_2_ siRNA or universal control did not (*F*_2,14_ = 7.48, *p* = .06; CRF_1_, *n* = 7; CRF_2_ siRNA, *n* = 5; universal control, *n* = 5). Further analysis revealed that microinjection of CRF_1_ siRNA suppressed the expression of CRF_1_‐LI in the NG compared with that in the CRF_2_ siRNA or the universal control siRNA injected NG (*p* = .009 and *p* = .004, respectively, Figure 3).

### 3.5 Subdiaphragm vagotomy

**FIGURE 4:**
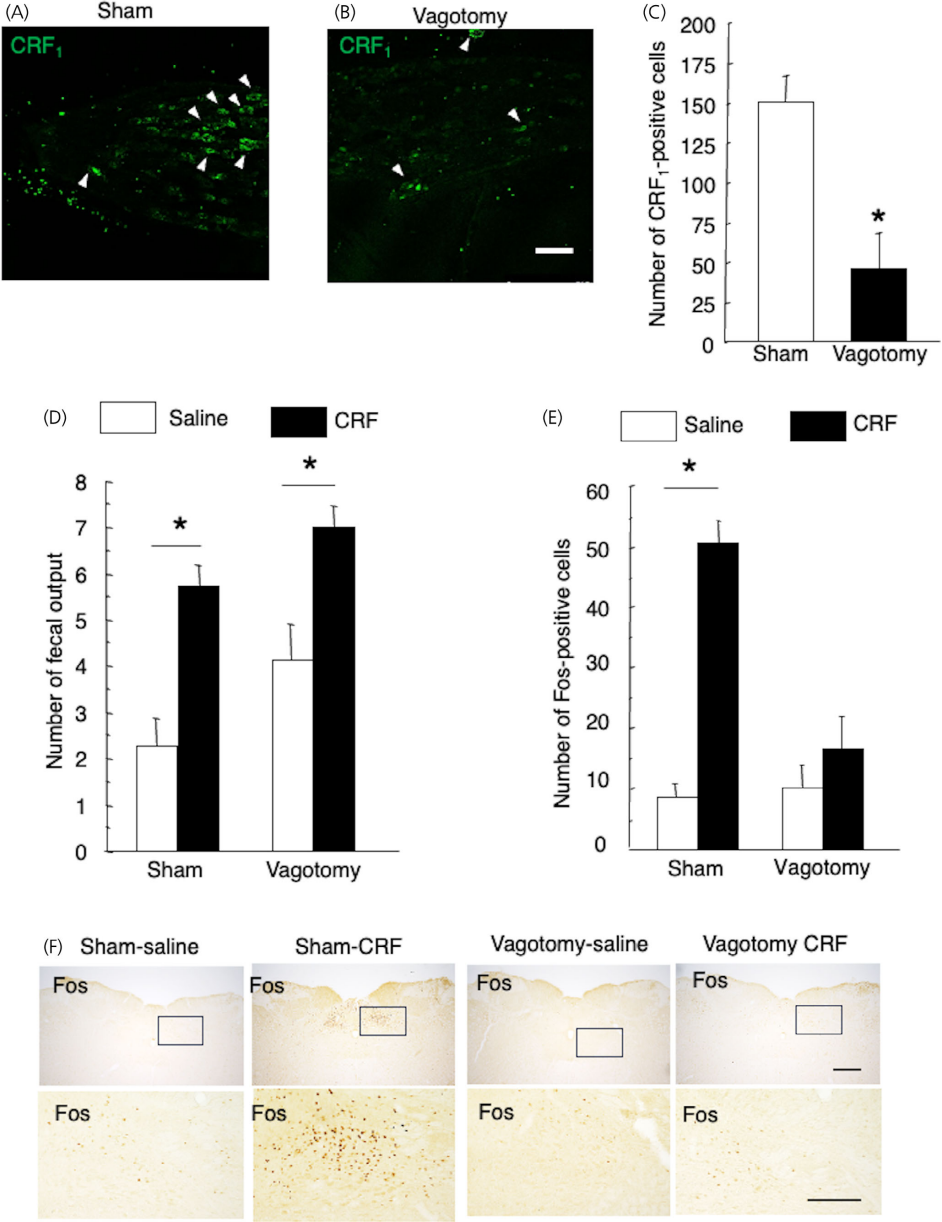
Effects of subdiaphragmatic vagal nerve transection on corticotropin‐releasing factor type 1 receptor‐like immunoreactivity (CRF_1_‐LI) in the nodose ganglion (NG) and intraperitoneal injection of corticotropin‐releasing factor (CRF) on the fecal output and Fos expression in the nucleus tractus solitarius (NTS). Number of CRF_1_‐positive cells in vagotomized or sham‐operated rats. Representative images of the immunohistochemical analysis of CRF_1_‐LI in the NG of sham‐operated (A) and vagotomized (B) rats, and data are shown in C. Data are presented as means ± SEM. Scale bar = 100 μm. White arrowheads indicate CRF_1_‐positive cells. *p < .01 when compared with sham‐operated rats. Number of CRF‐induced increasing fecal output on sham‐operated rats with saline (sham‐saline group), sham‐operated rats with CRF (sham‐CRF group), vagotomized rats with saline (vagotomy‐saline group), and vagotomized rats with CRF (vagotomy‐CRF group) (D). Data are presented as means ± SEM (sham‐saline, *n* = 8; sham‐CRF, *n* = 7; vagotomy‐saline, *n* = 8, and vagotomy‐CRF, *n* = 8). Number of Fos‐positive cells in the NTS in the same experiment groups (E). Data are presented as means ± SEM. Each experimental group contained four rats. Representative images show the Fos expression in the NTS (F) in the experiment group. Scale bar in the upper panels = 200 μm. The lower panels indicated high magnification of the boxed area in the upper panels, respectively. Scale Bar = 50 μm. *p < .01 when compared with saline.

The number of CRF_1_‐positive cells in the NG of vagotomized rats decreased compared with that of sham‐operated rats (vagotomized rats vs. sham‐operated rats, 45.5 ± 13.9 vs. 149.7 ± 24.6, *p* = .004, *n* = 6 in each group) (Figure 4A–C).

No significant interaction was found between vagotomy and CRF injection; however, a significant difference was observed in the effect of CRF injection on fecal output (*F*_1,27_ = 26.9, *p* = .001; sham‐saline, *n* = 8; saline‐CRF, *n* = 7; vagotomy‐saline, *n* = 8; vagotomy‐CRF, *n* = 8) (Figure 4D). Further analysis revealed that CRF increased fecal output with or without vagotomy relative to that in saline‐injected rats (*p* = .046 and *p* = .005) (Figure 4D).

A significant interaction was found between vagotomy and CRF injection on c‐Fos expression in the NTS (*F*_1,12_ = 18.4, *p* = .001, *n* = 4 in each group). Further analysis revealed that CRF increased c‐Fos expression in the NTS relative to saline‐injected sham‐operated rats (*p* < .0001). Vagotomy suppressed the CRF‐induced increase in c‐Fos expression in the NTS relative to saline‐injected vagotomized rats (Figure 4E,F).

### 3.6 Retrograde tracing

**FIGURE 5:**
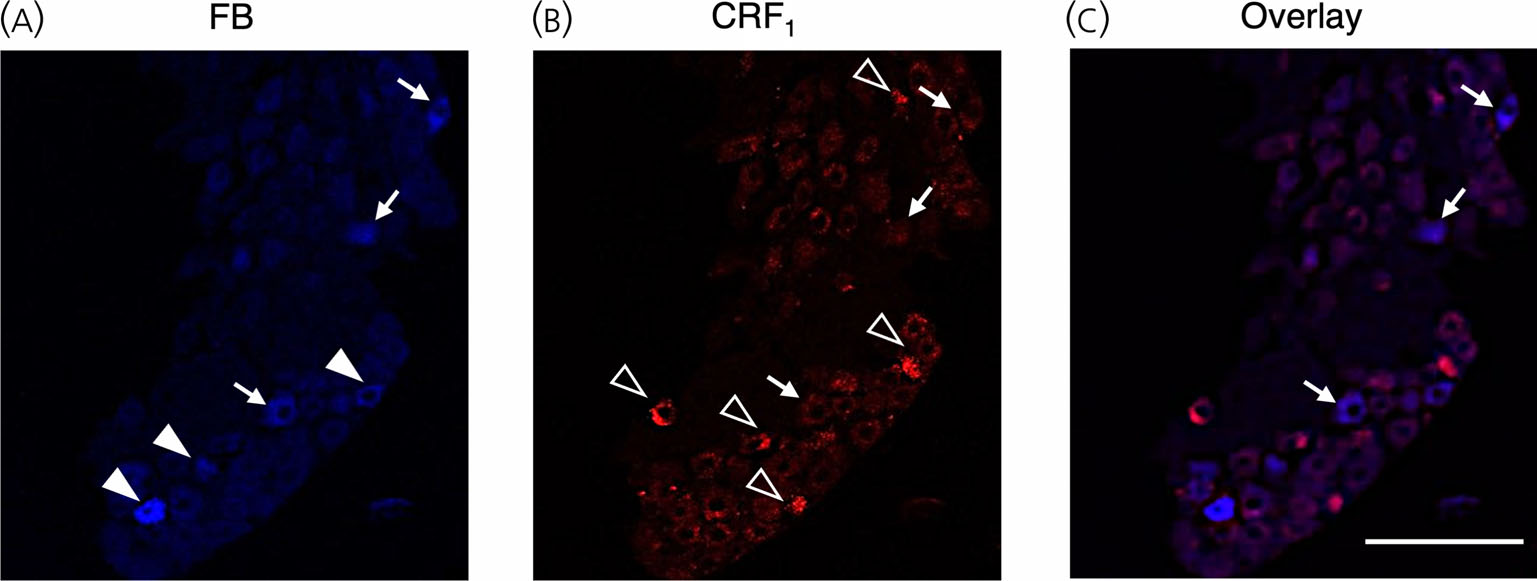
Retrograde neuronal tracing study. (A–C) Typical images of a retrograde tracer, fast blue (FB)‐labeled neurons (blue, left panel), corticotropin‐releasing factor type 1 receptor (CRF_1_)‐positive cells labeled with Alexa Fluor 647 (red, middle panel) and overlay images (right panel). The white arrows indicate dual‐labeled neurons, and the open arrowheads indicate only CRF_1_‐LI‐labeled cells, filled arrow heads indicate only FB‐labeled cells. Scale bar = 100 μm.

After injection of FB into the proximal colon, the FB‐positive cell count was 98 ± 12 per NG (*n* = 4 in each group); the percentage of CRF_1_ expression on FB‐positive cells was 57.8 ± 3.2% (*n* = 4) (Figure 5).

### 3.7 pCREB expression in the NG

**FIGURE 6:**
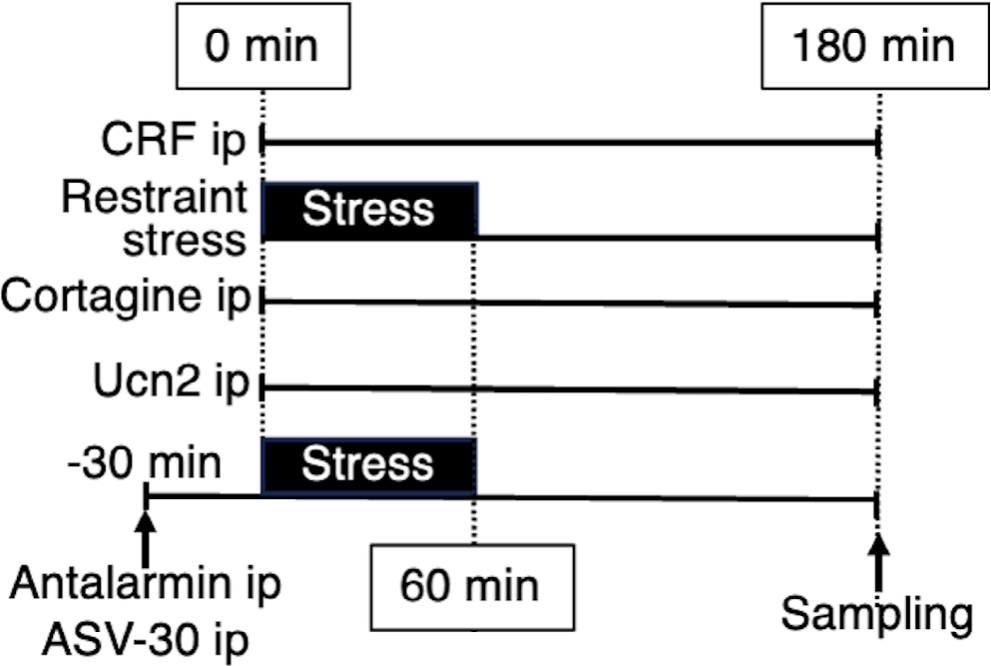
Timeline of drug injection and stress procedure. Corticotropin‐releasing factor (CRF), cortagine and urocortin 2 (Ucn 2) is intraperitoneally injected at 0 min of the timeline. Immobilization stress starts at 0 min and is released at 60 min, then stays in the home cage for 120 min. Intraperitoneal injection of antalarmin or antisauvagine‐30 (ASV‐30) conducts 30 min before the beginning of stress.

**FIGURE 7:**
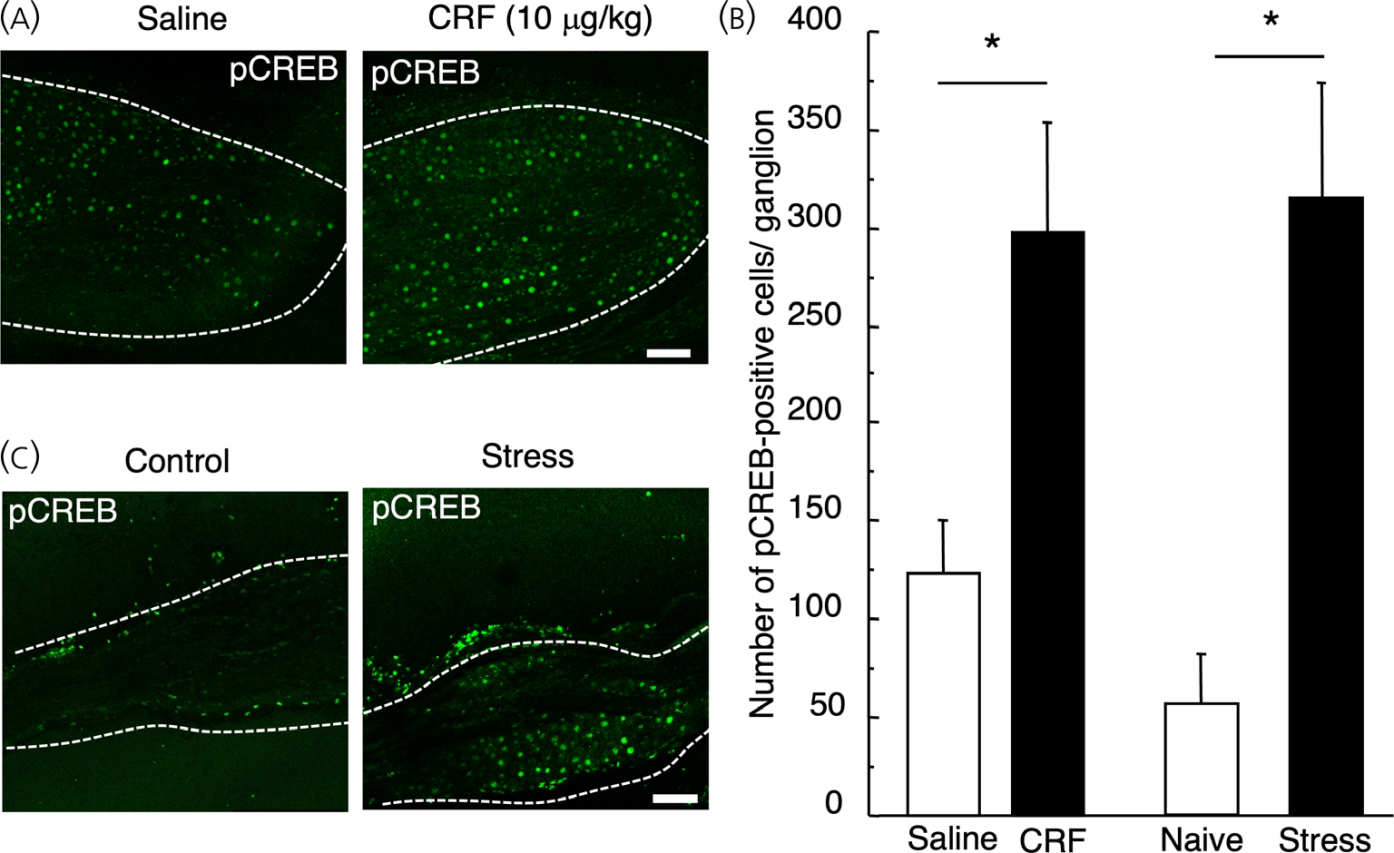
Effect of intraperitoneal corticotropin‐releasing factor (CRF) injection or immobilization stress on phospho‐cyclic AMP responsible element (pCREB) expression in the rat nodose ganglion (NG). Representative images of the immunohistochemical analysis of pCREB expression in the NG of saline‐injected rats, CRF (10 μg/kg)‐injected rats (A), naive rats, and stressed rats (C). Scale bar = 100 μm. Data are shown in (B) and presented as means ± SEM. *p < .01 when compared with control (saline vs. CRF, naive vs. stress).

A timeline of CRF injection and immobilization stress was shown in Figure 6. The intraperitoneal injection of CRF significantly increased pCREB expression in the NG (CRF‐injected rats vs. saline‐injected rats, 298.3 ± 55.4 vs. 124 ± 24.141, *p* = .012, *n* = 9 in each group, Figure 7A,B). Immobilization stress also significantly increased the expression of pCREB on vagal neurons (naive rats vs. stressed rats, 55.5 ± 25.9 vs. 311.8 ± 57.4, *p* = .002, *n* = 6 in each group, Figure 7B,C).

**FIGURE 8:**
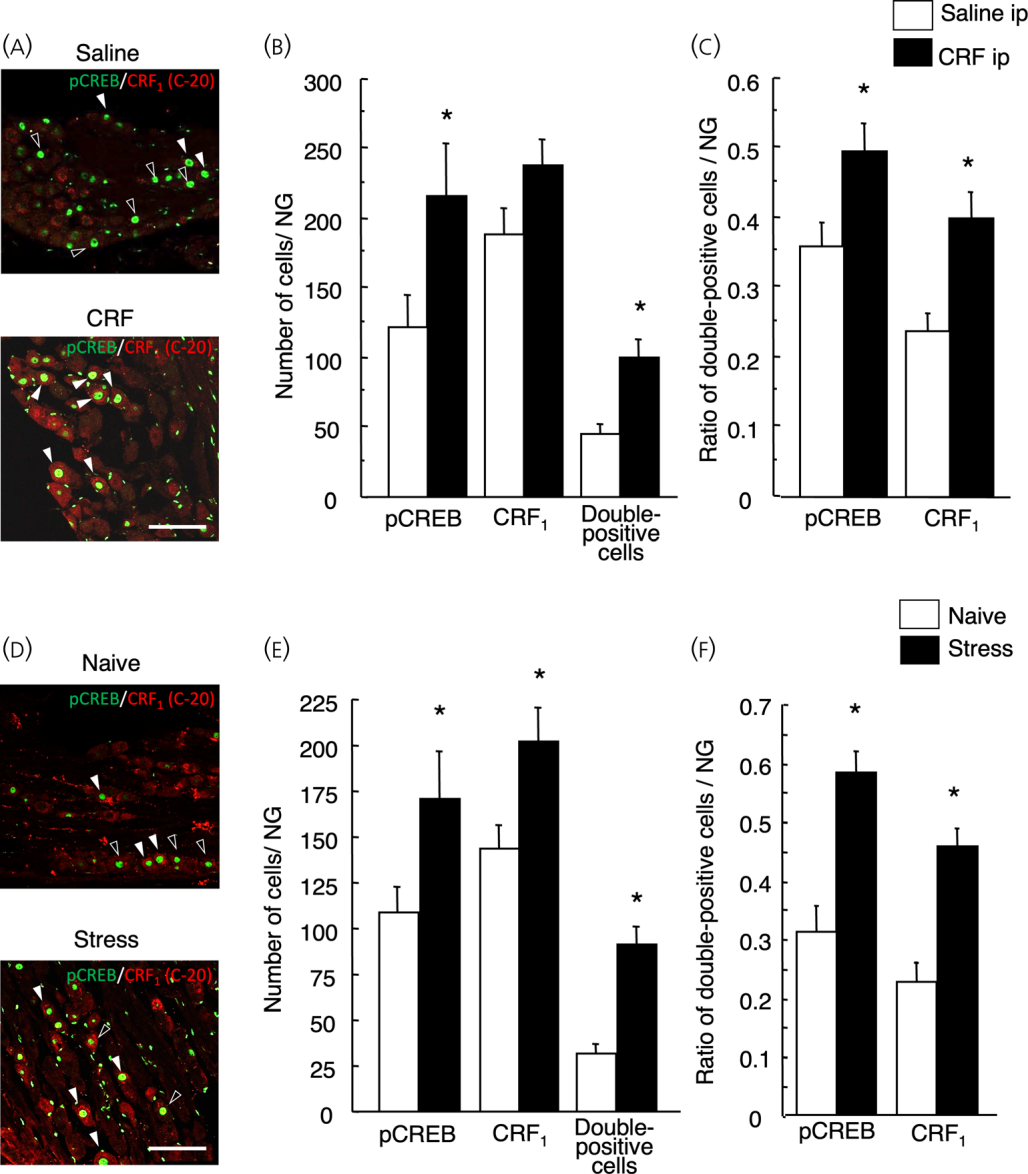
Effect of intraperitoneal corticotropin‐releasing factor (CRF) injection or immobilization stress on phospho‐cyclic AMP responsive element (pCREB) expression in the corticotropin‐releasing factor type 1 receptor (CRF_1_)‐positive cells in the nodose ganglion (NG). Representative images of the immunohistochemical analysis of pCREB (green) expression on the CRF_1_‐positive cells (red) in the NG of saline‐injected rats, CRF (10 μg/kg)‐injected rats (A). White arrowheads indicate pCREB expressing CRF_1_‐positive neurons (double positive cells) and black arrowheads indicate pCREB expressing CRF_1_‐negative neurons in the middle portion of the NG. Scale bar = 100 μm. Data of pCREB positive cells, CRF_1_‐positive cells, and pCREB expressing CRF_1_‐positive cells (double positive cells) are shown in (B) and presented as means ± SEM. Ratio of pCREB expressing CRF_1_‐positive cells (double positive cells) in pCREB expressing cells and in CRF_1_‐positive cells are shown in (C) and presented as means ± SEM. *p < .01 when compared with saline. Representative images of the immunohistochemical analysis of pCREB (green) expression on the CRF_1_‐positive cells (red) in the NG of naive rats and stressed rats (D). White arrowheads indicate pCREB‐expressing CRF_1_‐positive neurons (double positive cells) and black arrowheads indicate pCREB‐expressing CRF_1_‐negative neurons in the middle portion of the NG. Scale bar = 100 μm. Data of pCREB‐positive cells, CRF_1_‐positive cells, and pCREB‐expressing CRF_1_‐positive cells (double positive cells) are shown in (E) and presented as means ± SEM. The ratio of pCREB‐expressing CRF_1_‐positive cells in pCREB‐expressing cells (double positive cells) and in CRF_1_‐positive cells is shown in (F) and presented as means ± SEM. *p < .01 when compared with naive rats.

Next, we investigated whether intraperitoneal CRF injection or immobilization stress induced pCREB expression in CRF_1_‐positive cells (Figure 8). Intraperitoneal injection of CRF increased the expression of pCREB in CRF_1_‐positive cells (CRF‐injected rats vs. saline‐injected rats, 99.6 ± 13.2 vs. 44.8 ± 7.8, *p* = .04, *n* = 13 in each group, Figure 8A,B). It also increased the ratio of pCREB expressing CRF_1_‐positive cells in CRF_1_‐positive cells (CRF‐injected rats vs. saline‐injected rats, 0.50 ± 0.04 vs. 0.36 ± 0.03 *p* = .002, *n* = 13 in each group, Figure 8C) and in pCREB expressing cells in the NG relative to that of saline (CRF‐injected rats vs. saline‐injected rats, 0.40 ± 0.04 vs. 0.24 ± 0.03 *p* = .015, *n* = 13 in each group, Figure 8C).

Immobilization stress also increased the expression of pCREB in CRF_1_‐positive cells (stressed rats vs. naive rats, 91.25 ± 9.5 vs. 31.8 ± 4.8, *p* < .0001, stressed rats, *n* = 12; naive rats, *n* = 10; Figure 8D,E), CRF_1_‐positive cells (stressed rats vs. naive rats, 202.2 ± 18.1 vs. 143.6 ± 12.7, *p* = .019, stressed rats, *n* = 12; naive rats, *n* = 10; Figure 8E), and the ratio of pCREB expressing CRF_1_‐positive cells in CRF_1_‐positive cells (stressed rats vs. naive rats, 0.58 ± 0.04 vs. 0.31 ± 0.05, *p* = .033, stressed rats, *n* = 12; naive rats, *n* = 10; Figure 8F) and in pCREB expressing cells in the rat NG relative to that of naive rats (stressed rats vs. naive rats, 0.46 ± 0.03 vs. 0.23 ± 0.03, *p* ≤ .0002, stressed rats, *n* = 12; naive rats, *n* = 10; Figure 8F).

### 3.8 The effect of CRF_1_ agonist, Ucn 2, CRF_1_ antagonist, and CRF_2_ antagonist on the stress‐induced pCREB expression

**FIGURE 9:**
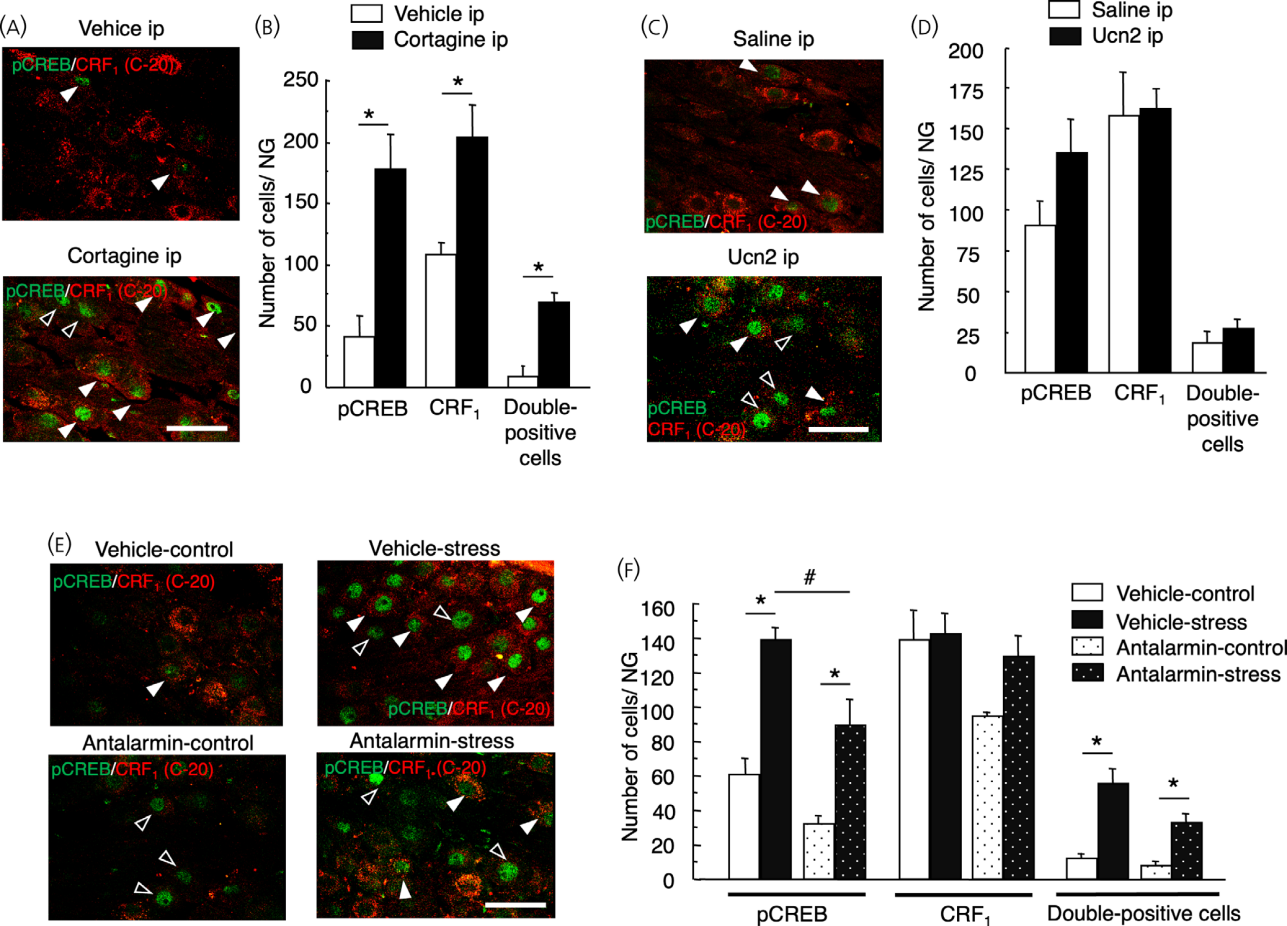
Effect of intraperitoneal cortagine and urocortin 2 (Ucn 2) on phospho‐cyclic AMP responsive element (pCREB) expression in the corticotropin‐releasing factor type 1 receptor (CRF_1_)‐positive cells in the nodose ganglion (NG). Representative images of the immunohistochemical analysis of pCREB (green) expression on the CRF_1_‐positive cells (red) in the NG of vehicle‐injected rats, cortagine (10 μg/kg)‐injected rats (A) and Ucn 2 (10 μg/kg)‐injected rats (C). White arrowheads indicate pCREB‐expressing CRF_1_‐positive neurons (double positive cells) and black arrowheads indicate pCREB‐expressing CRF_1_‐negative neurons in the middle portion of the NG. Scale bar = 50 μm. Data of pCREB‐positive cells, CRF_1_‐positive cells, and pCREB‐expressing CRF_1_‐positive cells (double positive cells) are shown in (B) and (D) and presented as means ± SEM. *p < .01 when compared with vehicle. Effect of intraperitoneal antalarmin on stress‐induced pCREB expression in the CRF_1_‐positive cells in the NG. Representative images of the immunohistochemical analysis of pCREB (green) expression on the CRF_1_‐positive cells (red) in the NG of vehicle‐injected non‐stressed rats, vehicle‐injected stressed rats, antalarmin (20 mg/kg)‐injected non‐stressed rats, and antalarmin (20 mg/kg)‐injected stressed rats (E). White arrowheads indicate pCREB‐expressing CRF_1_‐positive neurons (double positive cells) and black arrowheads indicate pCREB‐expressing CRF_1_‐negative neurons in the middle portion of the NG. Scale bar = 50 μm. Data of pCREB‐positive cells, CRF_1_‐positive cells, and pCREB‐expressing CRF_1_‐positive cells (double‐positive cells) are shown in (F) and presented as means ± SEM. *p < .01 when compared with vehicle in the same group of stress status; ^#^ *p* < .01 when compared with antalarmin‐treated stressed rats.

The intraperitoneal injection of cortagine significantly increased pCREB expression in the NG (cortagine‐injected rats vs. vehicle‐injected rats, 178.0 ± 38.1 vs. 33 ± 11.4, *p* = .01), CRF_1_‐positive cells (cortagine‐injected rats vs. vehicle‐injected rats, 205 ± 32.1 vs. 94.8 ± 4.1, *p* = .034), pCREB expression in the CRF_1_‐positive cells (cortagine‐injected rats vs. vehicle‐injected rats, 69.8 ± 5.9 vs. 8.3 ± 3.3, *p* < .0001, *n* = 4 in each group, Figure 9A,B).

The intraperitoneal injection of Ucn 2 did not increase pCREB expression, CRF_1_‐positive cells, or pCREB expression in the CRF_1_‐positive cells (saline, *n* = 4; Ucn 2, *n* = 5, Figure 9C,D).

No significant interaction was found between drug and stress; however, a significant difference was observed in the effect of antalarmin and the effect of stress on pCREB expression (*F*_1,13_ = 25.1, *p* = .0002, *p* < .0001 respectively). Further analysis revealed that pCREB expression in the NG of antalarmin‐treated stressed rats was significantly lower than that of vehicle‐treated stressed rats (*p* < .02, vehicle‐naive, *n* = 4; vehicle‐stress, *n* = 5; antalarmin‐naive, *n* = 4; antalarmin‐stress, *n* = 4) (Figure 9E,F).

**FIGURE 10:**
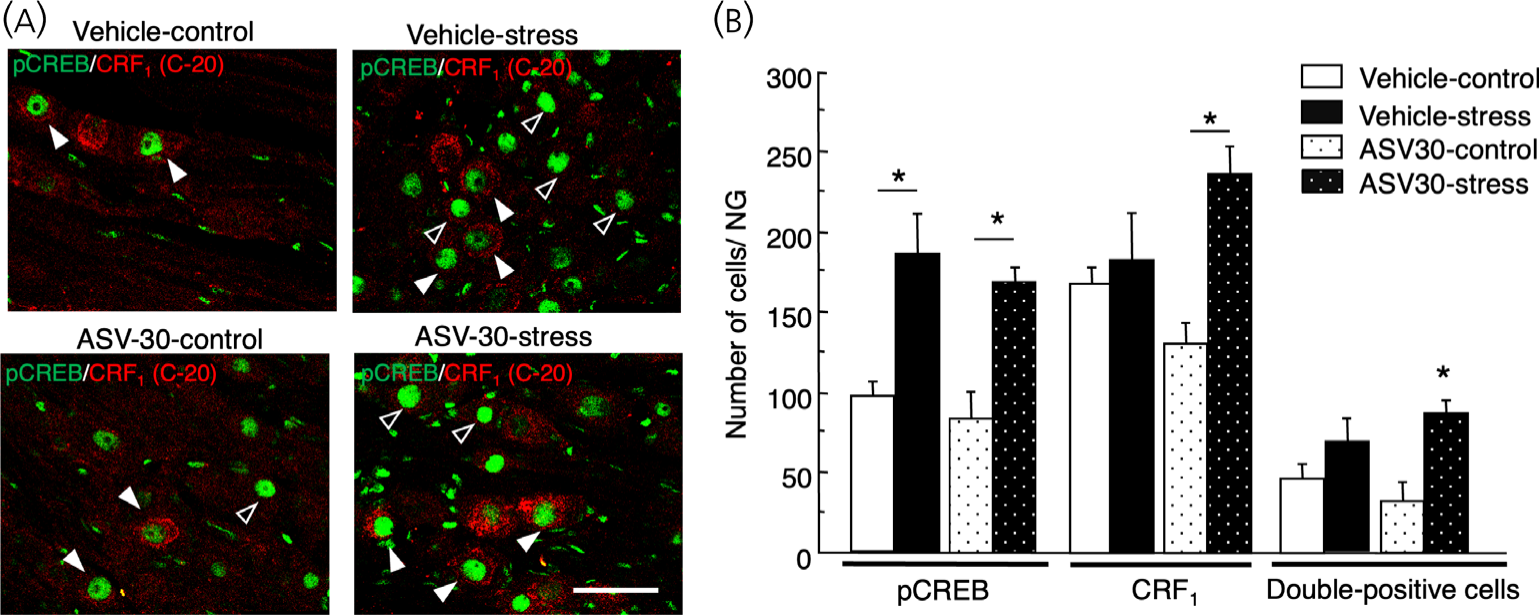
Effect of intraperitoneal antisauvagine‐30 (ASV‐30) on stress‐induced pCREB expression in the CRF_1_‐positive cells in the NG. Representative images of the immunohistochemical analysis of pCREB (green) expression on the CRF_1_‐positive cells (red) in the NG of vehicle‐injected non‐stressed rats, vehicle‐injected stressed rats, ASV‐30 (100 μg/kg)‐injected non‐stressed rats, and ASV‐30 (100 μg/kg)‐injected stressed rats (A). White arrowheads indicate pCREB‐expressing CRF_1_‐positive neurons (double positive cells) and black arrowheads indicate pCREB‐expressing CRF_1_‐negative neurons in the middle portion of the NG. Scale bar = 50 μm. Data of pCREB‐positive cells, CRF_1_‐positive cells, and pCREB‐expressing CRF_1_‐positive cells (double‐positive cells) are shown in (B) and presented as means ± SEM. *p < .01 when compared with vehicle in the same group of stress status.

No significant interaction was found between drug and stress; however, a significant difference was observed between the effect of stress on pCREB expression (*F*_1,13_ = 25.1, *p* = .0002). There was a significant interaction between drug and stress on the number of CRF_1_‐positive cells in the NG (*F*_2,13_ = 6.57, *p* = .024). Further analysis revealed that the number of CRF_1_‐positive cells in the NG of antisauvagine‐30‐treated stressed rats tended to be higher than that of vehicle‐treated stressed rats (*p* = .06), vehicle‐naive, *n* = 4; vehicle‐stress, *n* = 4; antisauvagine‐30‐naive, *n* = 5; antisauvagine‐30‐stress, *n* = 4 (Figure 10).

## 4 DISCUSSION

This study provides evidence that CRF_1_‐LI is widely distributed in the cholinergic vagal afferent neurons and is influenced by subdiaphragmatic vagotomy. Through a retrograde tracing study, we clarified that some vagal afferent neurons terminating at the proximal colon expressed CRF_1_‐LI. This is the first study to show that vagal afferent neurons respond to intraperitoneal CRF injections or immobilization stress. These results contribute to a better understanding of the role of NG in transducing visceral sensory information, which may be activated by the CRF/CRF_1_ system under stressful conditions.

Using anti‐CRF_1_ antiserum, we for the first time showed that CRF_1_‐LI was distributed throughout the NG, as previously reported for CRF_1_‐LI expression in the pituitary gland.^20^ Dual immunofluorescence analysis revealed that the distribution of CRF_1_‐LI detected with anti‐CRF_1_ antiserum was nearly the same as that of CRF_1_‐LI detected with the commercially available antiserum against CRF_1_ (C‐20). The C‐20 antiserum is prepared by immunization with a fragment corresponding to the C‐terminus of CRF_1_, which is highly homologous to CRF_2_, and its specificity is not high. The specificity of our anti‐CRF_1_ antiserum used here was already proven.^20^

### 4.1 Western blot analysis of CRF_1_ from rat NG

Western blot analysis of tissue extracts from the rat NG and pituitary gland showed that the anti‐CRF_1_ antiserum detected bands at 65, 58, 48, and 37 kDa. The 48 kDa band is corresponding to the non‐glycosylated form of CRF_1_. Previously, we reported that bands were detected in the rat pituitary, and that they were not present when the antiserum was pretreated with the antigenic CRF_1_ fragment, but not the 37‐kDa band. These bands were present when the antiserum was pretreated with the CRF_2_ fragment corresponding to the antigenic CRF_1_ fragment.^20^ Immunohistochemical analysis revealed that CRF_1_‐LI in the pituitary was completely abolished when anti‐CRF_1_‐antiserum was pre‐treated with CRF_1_ antigenic peptide,^20^ so the protein fragment of 37 kDa may be produced in the sample preparation process for western blotting. In our previous study, western blot analysis of extracts from HEK‐293 cells, which expressed EGFP‐tagged CRF_1_ transfectants, detected bands that corresponded to EGFP‐tagged CRF_1_, using the anti‐CRF_1_ antiserum. Furthermore, western blot analysis showed that the addition of siRNA against CRF_1_ to primary cultured rat anterior pituitary cells suppressed the expression of the band of CRF_1_. Based on the amino acid sequence, the estimated molecular weight of CRF_2_ is ~50 kDa, and the band of 48 kDa in Figure 1 was a single line and a narrow band, so the antiserum specifically recognizes CRF_1_ (48 kDa) and not CRF_2_. The evidence also shows that anti‐CRF_1_ antiserum specifically recognizes CRF_1_ not CRF_2_.^20^

### 4.2 Validation of CRF_1_ ‐LI in the rat NG

As shown in the present study, CRF_1_‐LI was not detected with the antiserum pretreated with the antigenic fragment, but was comparable with the antiserum that pretreated with the CRF_2_ fragment corresponding to the antigenic portion of CRF_1_. CRF_1_‐siRNA significantly suppressed the expression of CRF_1_‐LI in the NG, compared with the CRF_2_‐siRNA and the universal control siRNA. The result suggested that CRF_1_‐LI antiserum specifically recognizes CRF_1_ not CRF_2_. These results indicate that the anti‐CRF_1_ antiserum is specific to CRF_1_.^20^

Vagal nerve trunk ligation on the peripheral side of the NG induced CRF_1_‐LI accumulation on the proximal side of the ligated nerve trunk. It has been suggested that anterograde transport is consistent with the synthesis of receptor peptides in the cell body and is transported to the site of action^32^; therefore, the protein accumulates proximal to the ligature.^33^ These results revealed that CRF_1_ was transported from the NG cell bodies toward the peripheral sensory terminals. Because the peripheral process of the vagal nerve contains both motor and sensory fibers, further studies are needed to clarify whether transport occurs by the selection of afferent or efferent fibers, or in combination.

In a previous study, the expression of CRF_2_ mRNA is detected in rat NG using RNA sequencing, and the expression of CRF_2_ mRNA is elucidated by in RNA scope.^15^ The expression of CRF_1_ in the mice NG was reported to be within a noise level in the study,^34^ suggesting that CRF_1_ is not detected in the mice NG. Single‐cell analysis also revealed that the expression of CRF_2_ mRNA is much higher than that of CRF_1_ in the mice NG.^35^ Then, we examined the expression levels of CRF_1_ and CRF_2_ mRNA using RT‐qPCR in the rat NG and demonstrated that the expression of CRF_1_ transcript variant 1 is detected in the rat NG, although the expression levels are lower than those of CRF_2_ mRNA. The results of the present study indicated, for the first time, the expression of CRF_1_ mRNA and immunoreactivity in situ in rat NG.

Dual‐immunofluorescence studies showed that most CRF_1_‐positive cells expressed ChAT, which is the enzyme that catalyzes the biosynthesis of acetylcholine, whereas approximately half of the ChAT‐positive cells expressed CRF_1_. The expression of ChAT is consistent with the enzyme's function in generating presynaptic stores of acetylcholine. A previous study indicated that almost all neurons in the NG are cholinergic.^36^ Activation of vagal afferent neuronal cell bodies in the NG may stimulate the release of acetylcholine from the central terminus of cholinergic neurons to the NTS. A previous study showed using patch‐clamp electrical experiments that nicotine and cytisine, α7 nicotinic agonist, have facilitating effects on glutamatergic neurotransmission of a neuron in the rat caudal NTS.^37^ These investigations suggest that the activation of CRF_1_‐positive cholinergic neurons may increase the release of acetylcholine into the NTS via nerve terminals. Thus, CRF_1_‐positive cells may be involved in the regulation of CRF‐induced visceral sensation through the transmission of cholinergic vagal afferent neurons.

### 4.3 Subdiaphragmatic vagotomy

Vagal afferent neurons innervate internal organs such as the heart, lungs, and gastrointestinal tract.^38^ Thus, we examined the effect of subdiaphragmatic vagotomy on the number of CRF_1_‐positive neurons in the NG, intraperitoneal injection of CRF‐induced fecal output, and c‐Fos expression, a marker of neuronal activity, in the NTS in the brainstem.

The number of CRF_1_‐positive neurons in the vagotomized rats decreased to one‐third of that in the sham‐operated rats, suggesting that two‐thirds of the CRF_1_‐positive neurons in the NG innervate subdiaphragmatic organs, such as the stomach, small intestine, colon, liver, and pancreas. CRF is involved in stress‐induced increases in colonic transients. There is evidence that peripheral injection of CRF acts directly on the colon through activation of enteric and endocrine cells releasing 5‐HT.^9^, ^30^, ^39^, ^40^ In this study, intraperitoneal CRF‐induced fecal output was not affected by subdiaphragmatic vagotomy in accordance with a previous report.^31^ Antalarmin, a non‐peptide CRF_1_ antagonist, injected intraperitoneally abolishes CRF‐induced fecal output.^31^ An in vitro experiment shows that CRF directly stimulates the electrical activity and motility of rat‐isolated colonic distal segments, and these CRF effects are antagonized by a specific CRF antagonist α‐helical CRF_9–41_.^41^ The intraperitoneal injection of CRF stimulates colonic motor functions such as motor index and fecal output, and both CRF_1_/CRF_2_ antagonist astressin, and CRF_1_ antagonist CP‐154, 526 blocks these actions; thus, the colonic response to CRF is mediated by CRF_1_.^7^, ^42^ The receptor‐mediated action of intraperitoneal CRF on stimulating colonic motor function likely occurs at peripheral sites. A pharmacokinetic study established that CRF is not effectively transported from the blood to the brain.^43^ These studies suggest that peripheral CRF/CRF_1_ may participate in stress‐induced gastrointestinal dysfunction.

Two distinct receptor subtypes of CRF, CRF_1_, and CRF_2_, exhibit opposing functions to CRF and stress. CRF_2_ antagonist astressin‐2B is reported to enhance the colonic contraction response induced by CRF. Ucn 2, an endogenous ligand of CRF_2_, blocks the increasing colonic contraction response to CRF.^42^ In an in vitro study, CRF increases the contractions of colonic muscle strips, and Ucn 2 does not alter the contractions but blocks CRF.^42^ Thus, colonic CRF_2_ may play a modulatory role in the CRF/CRF_1_ signaling mediating the regulation of colonic functions. Ucn 2 does not alter the contractions but blocks the stimulatory action of CRF^11^, ^42^; therefore, CRF_2_ may modulate CRF/CRF_1_ signaling during colonic motor activity.

In the NTS, intraperitoneal CRF‐induced c‐Fos expression was not increased by vagotomy. Since the afferents of the subdiaphragmatic vagal nerve project to the NTS in the brainstem,^44^ the results show that visceral information induced by intraperitoneal CRF injections may be transduced to the NTS via vagal afferents. The inhibitory effect of vagotomy was certainly significant, although colonic sensory information includes not only vagal afferents but also spinal afferents via the dorsal root ganglion in mice.^45^ In the present study, vagotomy did not affect defecation involving the distal colon. A previous study reports that the number of c‐Fos‐positive myenteric neurons induced by intraperitoneal CRF injection is the same in both the proximal and distal colons of rats,^39^ and the expression of CRF_1_ is much lower in the distal colon than in the proximal colon.^46^ Moreover, vagal afferents project to the proximal colon rather than to the distal colon in mice.^47^ Vagus innervates mainly the rat proximal stomach, and peripheral injection of CRF markedly inhibits gastric emptying and contractions, which are partly blocked by vagotomy.^48^ In rats, fos expression in the NTS induced by gastric distension as a satiety signal is blocked by vagotomy.^49^ Thus, gastric sensory information induced by CRF may not induce fos expression in the NTS. The inhibitory effect of CRF on gastric movement is blocked by the CRF_2_ antagonist but not by CRF_1_.^10^, ^50^ These findings suggest that intraperitoneal CRF injection may cause increasing colonic contraction via CRF_1_ and that sensory information may be transmitted to the NTS from vagal afferent neurons rather than from spinal afferent neurons. The results confirmed from vagotomized rats also indicated the importance of mesenteric CRF–responsible organ–NG interactions, and that vagal afferent neurons may play a crucial role in transducing peripheral CRF signals to the central nervous system.

### 4.4 Retrograde tracing and axonal transport

Since previous studies show that the vagal innervation of the colon becomes sparser in the distal colon than in the proximal colon,^45^, ^47^ FB was injected into the proximal colon within 5 cm distal to the cecocolic junction in the present study. The retrograde tracing of the FB clarified that approximately half of the FB‐positive neurons expressed CRF_1_ in the NG. The neuronal tracing study also indicated that the number of FB‐labeled cells in the NG was ~97 per NG, and the whole vagal ganglia contained 1277 ± 98 neurons. The proportion of nodose neurons innervating the proximal colon in the NG is ~7.6%. A previous study using the retrograde lipophilic tracers DiI and DiO injected into the rat distal colon demonstrates that each NG contains ~179 labeled afferents.^51^ Another tracing study evaluates the injection of Alexa Fluor 488 cholera toxin subunit B into the serosal layer of the proximal colon in mice, labeling ~96 nodose neurons.^45^ Unfortunately, neither retrograde study calculated the percentage of labeled neurons among all nodose neurons. To the best of our knowledge, this is the first study to determine the percentage of CRF_1_‐positive neurons innervating the proximal colon in the NG neurons. Recently, the right NG has a higher number of neurons than the left innervating the proximal colon.^47^ It is possible that there is a difference in CRF_1_‐LI between the right and left, and further observation revealed it.

The results of this retrograde tracing study revealed that ~58% of the vagal neurons that innervate the area of the proximal colon injected with FB were CRF_1_‐positive. In a previous study, 5% true blue is injected into the ventral wall of the rat proximal colon and 50% of retrogradely labeled nodose neurons are positive for neuropeptide Y type 2 receptor (Y2R).^29^ However, the function of colon‐innervated Y2R‐positive neurons remains unclear. Although the function of colon‐innervated CRF_1_‐positive neurons is unclear, their role can be anticipated in the transmission of CRF‐induced sensory information to the central nervous system. CRF_1_‐positive neurons in the NG may be required to assess the relationship between the central vagal nuclei and the proximal colon involved in a stress‐induced visceral event.

### 4.5 Stress‐induced pCREB expression in the NG

This study demonstrated for the first time that intraperitoneal CRF or immobilization stress increased pCREB expression in rat NG. CRF‐ or stress‐induced increase in pCREB expression in CRF_1_‐positive vagal afferent neurons is also considered novel; however, the mechanism is unclear. The present study revealed that cortagine, CRF_1_ agonist, induced pCREB expression in the NG, and pretreatment with antalarmin, CRF_1_ antagonist, suppressed immobilization stress‐induced increase in pCREB expression in the NG. These results demonstrated that CRF_1_ is involved in the stress‐induced NG activation.

A previous study shows intraperitoneal injection of CCK8 induces pCREB expression in cannabinoid type 1 receptor‐positive neurons in the rat NG^23^; thus, peripheral information is thought to be detected by vagal afferent neurons and transduced into the central nervous system.^50^ Similarly, information on visceral senses, such as increased colonic motility and decreased colonic transit and time after transit, induced by intraperitoneal CRF or immobilization stress seems to be transmitted from the colon to the brain via CRF_1_‐positive vagal afferent neurons expressing pCREB. CRF‐ or stress‐induced pCREB‐expressing CRF_1_‐positive neurons are not limited to colonic innervation. Stress‐induced CRF from enteric neurons, enteric immune cells, and mucosal enterochromaffin cells^12^ may act at CRF_1_ on vagal afferent nerve terminals and cause membrane depolarization and action potential discharge in the NG. Consequently, the action potential induces the activation of intracellular signaling and the expression of pCREB in the NG. Moreover, direct interaction between circulating CRF and CRF_1_ in the NG seems to induce pCREB expression.

Immobilization stress increases the population of CRF_1_‐positive cells, whereas intraperitoneal injection of CRF did not. A previous study indicates that the intraperitoneal injection of CCK‐8 induces an increase in the population of Y2R‐positive neurons in the rat NG, 2–3 h after injection.^29^ However, the mechanism through which CCK‐8 induces an increase in the population of Y2R‐positive cells remains unclear. It is possible that the axonal transport of Y2R may be inhibited, leading to Y2R‐LI accumulation in the cell body. Otherwise, transcription factors may be rapidly activated by extracellular signals, resulting in accelerated receptor synthesis. Similar mechanisms may occur during stress‐induced increases in CRF_1_‐positive cell populations. Interestingly, antisauvagine‐30, a CRF_2_ antagonist, tended to enhance stress‐induced increases in the population of CRF_1_‐positive cells. This suggests that a similar mechanism exists whereby the CRF_2_ receptor inhibits the action of the CRF_1_ receptor in the colon under stress conditions.^11^

Microinjection of CRF into the proximal colon did not induce pCREB expression in the NG under anesthesia (data not shown). Because direct microinjection of CRF into the intestinal wall was performed under anesthesia, appropriate arousal conditions may be required for signal transduction from peripheral organs to the NG. Since the stress response is influenced by sex hormones,^21^ it is expected that stress‐induced activation of the NG would also be affected by sex hormones. Further experiments using female rats are required to clarify the influence of sex hormones on the NG activation in stress.

## 5 CONCLUSION

This study demonstrates the possible role of the CRF/CRF_1_ system in the signal transduction of colonic sensory information to the central nervous system via the NG, and suggests that stress‐induced vagal activity is probably mediated by CRF_1_.

## AUTHOR CONTRIBUTIONS

**Asuka Mano‐Otagiri:** Writing – original draft; writing – review and editing; conceptualization; methodology; software; data curation; investigation; validation; formal analysis; funding acquisition; project administration; resources; visualization. **Tamotsu Shibasaki:** Writing – review and editing. **Atsushi Sakai:** Methodology. **Yoshihiko Kakinuma:** Writing – review and editing; funding acquisition; methodology; project administration; supervision; resources.

## FUNDING INFORMATION

This study was supported in part by Grant‐in‐Aid for Scientific Research of Japan (Grant Number JP23K07023).

## CONFLICT OF INTEREST STATEMENT

The authors declare no conflicts of interest that would prejudice the impartiality of the work reported herein.

## PEER REVIEW

The peer review history for this article is available at https://www.webofscience.com/api/gateway/wos/peer-review/10.1111/jne.70082.

## Data Availability

The data that support the findings of this study are available from the corresponding author upon reasonable request.
